# Book Reviews

**Published:** 1816-02

**Authors:** 


### CRITICAL ANALYSIS

### *OF RECENT PUBLICATIONS*

IN THE

### DIFFERENT BRANCHES OF PHYSIC, SURGERY, AND MEDICAL PHILOSOPHY.

*Philosophical Transactions of the Royal Society of London, for the Year* 1815. Part II.

*Some additional Experiments and Observations on the Relation which subsists between the Nervous and Sanguiferous Systems;* by A. P. Wilson Philip, Physician at Worcester. Communicated by T. Andrew Knight, Esq. F. R. S.

In the first of these experiments it was found, that, when the spinal marrow of a frog was destroyed, by moving in various directions a wire passed through a hole in the lowest part part and up to the brain, that still the circulation was continued in the web.

"The labours of M. le Gallois, (continues Dr. Philip) by ascertaining some facts of great importance, while others immediately connected with them escaped his observation, have involved the subject in such seeming contradictions as, at first view, to have persuaded me that some of his experiments were inaccurate. On repeating many of them, however, I was convinced of their accuracy. In some the destruction of the cervical part of the spinal marrow immediately destroyed the function of the heart; yet in others the destruction, in a different way, of the same, or a larger portion of the spinal marrow, little affected it. In some, the greater part of the spinal marrow was destroyed without destroying the function of the heart; yet in others, after the spinal marrow had been divided, he found the function of the heart destroyed by the destruction of either half.

"It was the confusion arising from these, and similar difficulties, that occasioned him to observe that he had almost as many results as experiments, and that he had resolved to abandon the investigation, when his explanation of the first of the foregoing difficulties, founded on the supposition which suggested the above experiment, presented itself to him. Had it occurred to him to compare this supposition with the latter difficulty, he would have doubted its accuracy.

"The seeming contradictions which appear in the experiments of M. le Gallois cannot be reconciled, except on principles different from those hitherto assumed by physiologists."

"It was evident in making the experiments related in that paper, that the laws which regulate the effects of stimuli applied to the brain and spinal marrow on the muscles of voluntary, and on those of involuntary motion, are very different. The following experiments point out more precisely in what this difference consists."

The next experiment shews the effect of passing a wire in different directions through the brain of a rabbit. The voluntary muscles were no way affected, excepting when the wire approached the extremities of the nerves, which the author calls their sources, whether in the brain or spinal marrow.

Another rabbit was rendered insensible by a blow on the occiput. After part of the cranium was removed and the thorax laid open, the heart was found regularly beating. It is remarkable that no mention is made of respiration, the only important circumstance connected with this part of the experiment. The wires were passed, as in the last experiment and with the same result.

In the third experiment, spirits of wine were applied, but, though in contact with the extremities of nerves, produced no convulsion.

In another rabbit, after a blow on the occiput, these experiments were repeated on the brain, and on opening the thorax the pulsation of the heart was increased more than by mechanical injury to the brain. Whether the experiment was made on the cerebrum or cerebellum the consequence was the same.

In the fourth experiment, a rabbit's head was cut off at the occiput; the limbs were for a time convulsed, which effect was renewed by the slightest touch on the cut end of the spinal marrow. Spirits of wine produced no such effects, but chemical stimuli; the strong acids induced powerful contractions. After a time these effects ceased on the voluntary muscles, but increased the action of the heart.

By the sixth experiment, it appears that, though the action of the heart may be increased by various stimuli, yet it is not rendered irregular, till the power of the organ is nearly destroyed.

By the seventh experiment, that, to produce convulsion in the voluntary muscles, the stimulus must be continued and even varied; but that this is not necessary to the increased pulsation of the heart, and that, as long as the stimulus is continued, so long the increased action remains, not being lessened as some physiologists assert, by continuing the application, excepting only when tobacco was used, whose stimulating property was very inconsiderable.

Having thus shewn that chemical stimuli applied to the nervous system excite the heart more than mechanical stimuli, and that the reverse takes place in the muscles of voluntary motion: that both kinds of stimuli excite the heart after they cease to have any effect on the above muscles, that the heart is stimulated by an application to any part of the brain and nervous system, whilst the voluntary muscles are only affected from the extremities of nerves; that these stimuli, whilst they increase, never render irregular the motion of the heart, though the voluntary muscles are drawn into the most irregular spasms; that the latter are chiefly affected on the immediate application of the stimulus, whilst the heart continues its increased action as long as the stimulating process continues:—the author concludes this part of his paper, assuring us, with much solemnity, that this difference in the effects produced on the muscles of voluntary and involuntary motion seems involved in much obscurity, and must be explained before we can be said to understand the relation between the nervous system and the heart.

We could wish on this occasion, that the author, whilst about his experiments, had examined some other muscles of involuntary motion besides the heart. All that he says concerning the heart, and much more, has been told us by Mr. Hunter, without the tediousness or disgust which attends the minute relation of experiments on living animals. We shall only quote two lines to teach Dr. Philip, that, before he repeats the tortures on frogs and rabbits, so justly censured by the celebrated fabulist, it might save them much misery and the valuable time of himself and the Royal Society, if he would see what has been done by others.

"The heart's motion (says Mr. Hunter) does not arise from an immediate impulse on the brain, as it does in the voluntary muscles." —*Treatise on the Blood*, p. 148.

In consulting this passage, Dr. P. will see to how little purpose all his remarks on the motion or quescence of the heart are, unless he attends to and describes the respiration at the same time.

But let us proceed to the rest of the paper, in which the author endeavours to trace the causes from which these differences (involved in so much obscurity) arise; and afterwards to ascertain whether the power of the blood-vessels, like that of the heart, is independent of the nervous system, or whether they are directly influenced by that system, or only through the medium of the heart.

"It appeared to me probable, from many experiments, that the cause of chemical stimuli, applied to the nervous system, producing a greater effect on the heart than mechanical stimuli do, is, that the former, from their nature, act on a larger portion of the brain and spinal marrow. If this opinion is correct, the mechanical stimulus will be rendered the most powerful by confining the chemical to a smaller space than the mechanical stimulus occupies.

"Exp. 8. Both in frogs and rabbits I applied to various parts of the brain and spinal marrow, and particularly to those parts from which the nerves originate, minute portions of strong spirit of wine, without at all influencing the action of the heart. When these small portions were applied to a great many parts, the heart began to beat more frequently. This of course was much the same thing as at once applying the spirit of wine to a larger part. We have seen in the foregoing experiments, that mechanical stimuli applied to any considerable portion of the nervous system, increase the action of the heart. It appears from the following experiments that we cannot affect the heart by mechanical stimuli confined to any small part either of the brain or spinal marrow."

The last paragraph is a little complicated; we conceive, however, that, when the spirit of wine was applied to one part at a time, the heart was not affected; but, to all at once or in a quick succession, that the heart began to beat more frequently. Somewhat similar effects were produced when mechanical stimuli were used in a similar way. Some other experiments experiments and remarks follow, from which the following conclusions are drawn:—

"That the heart is excited by all stimuli applied to any considerable part of the nervous system, while the muscles of voluntary motion are only excited by intense stimuli applied to certain small parts of this system.

"These facts being ascertained, the other differences observed in the effects of stimuli applied to the nervous system, on the heart and muscles of voluntary motion, are easily explained."

For this explanation, we must refer our readers to the paper, and offer our own, which is at least shorter.

In all injuries done to any part of the body, the heart sympathises, and the more so as the injury is the greater. Without looking for any other final cause, we know that the means of repairing an injury is inflammation, and that under inflammation in most cases, the motion of the heart is accelerated. But this increased action of the heart will often occur before we can discover any other marks of inflammation, and cease, or even become less than ordinary, when inflammation is excited in some particular parts. All this is well known without such cruel experiments.

Some remarks follow on the effects of communicating sensation by the nervous ganglia. In these there is nothing new.

We are now led to the experiments which are to ascertain, "whether the power of the blood vessels is as independent of the nervous system, as that of the heart; and whether this system possesses over them the same kind of influence, as over the heart."

Here it is agreeable to find that Dr. P. craves a little mercy for his suffering subjects.

"These experiments were made on the capillaries of the frog, which, from the extent and transparency of the web of its hind feet, and from its great tenacity of life, appeared the best subject for such experiments. It has been questioned, how far inferences drawn from experiments made on cold-blooded animals, can be supposed to apply to those of warm blood. Both Fontana and Dr. Monro observe, that in their experiments they found the system of both obeying the same laws. The experiments I have had occasion to lay before the society, tend to confirm this observation; and I may say the same of all the experiments I have made on both sets of animals. There are certain circumstances in which they evidently differ, in all others they seem to agree. The following experiments ought not to be unnecessarily repeated; and, as there is no part of the warm blooded animal on which they could be satisfactorily made except the mesentery, they would be attended with much greater suffering in this, than in the cold-blooded animal. Some of them, from the warm-blooded animal being less tenacious of life, could not be performed on it."

 This

This last remark is surely enough to shew that there is a difference in the economy of these two classes of animals. In the heart, this difference is the more striking, on account of the structure of their lungs, and the manner which cold-blooded animals can exist without continued respiration.

The succeeding experiment goes to shew that if a frog's head is cut off, and the spinal marrow destroyed, and the blood stopped by a ligature round the neck, that the circulation will for a time continue.—Did any one doubt it? or the inference, that the blood-vessels retain their power after the nervous system is destroyed.

The next experiment is to prove, that the vessels can support the motion of the blood independent of the heart. The experiments were all made on frogs, and are only remarkable for their unnecessary cruelty and unsatisfactory result, the economy of the animal being, as we observed, in whatever relates to respiration, different from the human.

We shall now offer the author's general conclusions, that our readers may see to what good purposes Dr. Philip has employed himself.

"From the foregoing experiments and observations, it appears,

"1. That the laws which regulate the effects of stimuli, applied to the nervous system, on the muscles of voluntary and involuntary motion, are different.

"2. That both mechanical and chemical stimuli, applied to any considerable portion of the nervous system, increase the action of the heart.

"3. That neither mechanical nor chemical stimuli applied to the nervous system, excite the muscles of voluntary motion, unless they are applied near to the origin of the nerves and spinal marrow.

"4. That mechanical stimuli applied to the nervous system, are better fitted to excite the muscles of voluntary motion, and chemical stimuli, than those of involuntary motion.

"5. That, after all, stimuli applied to the nervous system, fail to excite the muscles of voluntary motion; both mechanical and chemical stimuli, so applied, still excite the heart.

"6. That both mechanical and chemical stimuli applied to the nervous system, excite irregular action in the muscles of voluntary motion.

"7. That neither excite irregular action in the heart, nor is its action rendered irregular by sedatives, unless a blow which crushes the brain be regarded as a sedative.

"8. That the excitement of the muscles of voluntary motion takes place chiefly at the moment at which the stimulus is applied to the nervous system, that of the heart continues as long as the stimulus is applied.

"9. That the muscles of voluntary motion are excited by stimuli applied to very minute parts of the nervous system.

" 10. That no stimulus applied to any minute part of the nervous system, can excite the heart.

" 11. That the heart obeys a much less powerful stimulus than the muscles of voluntary motion.

" 12. That the facts expressed in the three last sentences 9, 10, 11, afford an easy explanation of those expressed in the preceding sentences.

" 13. That the power of the blood-vessels, like that of the heart, is independent of the nervous system.

" 14. That the blood-vessels can support the motion of the blood after the heart is removed.

" 15. That the blood-vessels are directly influenced through the nervous system in the same way that the heart is.

" 16. That analogous to what we observe in the heart, no stimulus or sedative applied to the nervous system, excites irregular action in the blood-vessels.

" 17. That the power of the blood-vessels, like that of the heart, may be destroyed through the nervous system.

" 18. That the office of the ganglia is to combine the influence of the various parts of the nervous system, from which they receive nerves, and to send off nerves endowed with the combined influence of those parts.

" 19. That the will has no influence over the muscles of involuntary motion, because in their ordinary action they obey stimuli, over which we have no influence, and because at all times we neither see, nor are otherwise conscious of, their motions; and consequently cannot direct them.

" 20. That we have reason to believe that the division of the encephalon into the cerebrum and cerebellum, relates to the sensorial functions, since it does not appear to relate to the nervous functions, the muscles of voluntary and those of involuntary motion being influenced in the same way by both.

" 21. That the sedative effect is not the consequence of previous excitement, but the effect of a certain class of agents."

It will be seen that we have omitted noticing some of the experiments, and consequently some of the results. We have, however, offered enough to shew the improvement our readers are likely to meet with by perusing the whole, which is the great object of reviewing.

---

*Physiological Researches on Life and Death;* by Xavier Bichat. Translated from the French, by F. Gold.

*(Concluded from our last.)*

In our last we gave as perspicuous a view as was consistent with our limits of the first part of this once popular work, concluding with one of those many physiological facts in which the accuracy and genius of Mr. Hunter has proved pre-eminent above all other philosophers.

The

The present division commences with "general considerations on death." Here we might have expected some notice of that most important distinction made by Mr. Hunter between absolute universal death and such a cessation of action as never can be restored. To the common reader the distinction may at first seem unimportant; but one who has repeated the experiments concerning digestion of the English and Italian physiologists, ought to have considered it maturely. Instead of this, we have no other division of death but sudden and gradual. The first comprehends injuries, apoplexies, and hæmorrhages in general; the second the more usual mode of death by old age or disease. It may be thought that the first division comprehends all that can be said concerning absolute universal death. The difference, however, will appear very evident, when, to use the author's words, after the death of the animal part, the organic part sometimes retains life. Thus, after all sensation from external objects has ceased, the circulation may remain. This, it is true, cannot continue long after respiration has ceased in red-blooded animals; yet, when respiration and circulation have ceased, it is well known that the peristaltic motion continues, and even the process of digestion. When these have ceased, still the parts remain susceptible of certain impressions. Of galvanism, perhaps, last of all. At length this also ceases, and putrifaction soon commences. Now, there are modes of dying in which sensation, respiration, circulation, the peristaltic motion, and even susceptibility to the galvanic influence, all cease at once. This is what Mr. Hunter very properly calls *absolute universal death*. The most certain means of producing this is by lightning. All other methods are uncertain; but a blow on the stomach, and the passions of the mind, are, after electricity, the most frequent causes. When this happens, a very sudden change takes place from high health, in which all the various functions are vigorous, to instant death in all the parts, with the cessation of action in all the organs. It is under these circumstances only that we find the stomach completely digested through its whole substance, because it is only under these circumstances that the gastric juice, secreted in full vigour, is applied to the dead stomach; and it is now universally admitted that the gastric juice can produce its effect only on dead matter. Having premised thus much, we shall proceed to an explication of the author's opinions on the various modes of death. These he divides according to the parts which die first. The parts most necessary to vitality he admits, with others, are the brain, the heart, heart, and the lungs. With this view, he concludes his introductory chapter as follows:

"We shall first enquire into those deaths which begin at the heart, and afterwards into those which begin in the lungs and in the brain. I shall explain in what way, when one of these organs is affected, the others die; and then demonstrate by what sort of mechanism the death of the various other parts of the body ensues. Lastly I shall determine, from the principles which I shall then have laid down, the nature of the different species of diseases which are peculiar to the heart, the lungs, and the brain."

Before we proceed, it is absolutely necessary that we should caution our readers against the inaccuracy of this language. In most cases we shall find that all these parts die at the same time, though it may often happen that action may cease in one before it ceases in either of the others. We must, therefore, in our own minds, always substitute cessation of action for death. We shall offer an example or two by way of illustration, after which we shall cease to interrupt the order, unless where there is an absolute necessity to point out the difference.

M. Bichat proceeds to consider the influence of the death of the heart over that of the brain. In this chapter he shows satisfactorily that the only influence of the heart on the brain is by means of its vessels. Cut, says he, the cardiac nerves, and the brain is no way affected, nay even the actions of the heart are not directly modified by that section. In considering the two sides of the heart, he prefers, instead of the division of right and left side, to speak of them as two hearts, namely, the red-blooded and the black-blooded. This is precisely following Mr. Hunter's language, who always described quadrupeds and red-blooded animals having lungs, as possessed of a double heart, or of "two hearts, the pulmonary and the corporeal." In dividing this part of the work, it is not a little curious to see how our author falls into the language we have recommended from Mr. Hunter. In section the first, the term death is dropped, and cessation of action substituted. "In what way," it is asked, "does the *cessation* of the *functions* of the red-blooded heart interrupt the functions of the brain?" It is hardly necessary to answer this question, as we well know that not only the brain, but every other organ, languishes and dies, when deprived of arterial blood. We wish, however, the author had omitted some fanciful remarks which so much disfigure the writings of the continental philosophers. Because the red blood is necessary for the brain, must it therefore follow that the nearer the heart is to the brain, the more sagacious the animal must be, and even that men with short necks are wiser wiser than men with longer necks? If there is any truth in this, it is certainly not sufficiently established to be admitted in a philosophical disquisition, and, what is more to the purpose, it makes no necessary part of the author's theories, or even of his proposed inquiries. It reminds us of Spurzheim's unfortunate remarks on the thick-necked animals being most salacious,—a remark which, had he confined it to the different form of the castrated from the entire animal of the same species, might have been admitted; but which, when extended to the possibility of inducing impotence by wounds in the neck, only shows us the impropriety of hinting at a theory the truth or fallacy of which should be reserved for demonstration.

As there is always an evident motion on the brain by the pulsation of the larger arteries, we are not disposed to dispute with the author whether this motion is necessary. We know it must exist as long as the heart beats, and that, when that ceases, death ensues. We shall therefore leave it to be determined by him and Sir Everard Home, who considers pressure as necessary to the brain.

The next section enquires, "In what manner does the cessations of the functions of the black-blooded heart interrupt the functions of the brain." This enquiry gives no adequate idea of the manner in which it is conducted. All the author's experiments go to show that blood extravasated on the brain by tying the jugulars, or air injected into the veins, is fatal; the first by inducing apoplexy, the latter by the high irritation it excites in the brain, as is proved by the consequent convulsions. In showing that the brain and not the heart is principally affected, the author again falls into the correct language of Mr. Hunter: in short, he does so whenever he finds himself under the necessity of explaining himself with peculiar perspicuity, in consequence of his engaging in controversy.

"Now, (says he,) what is the organ so readily affected by the contact of air? I affirm it to be the brain, and not the heart; and maintain that the circulation is annihilated only because the cerebral *actions* have previously been so.

"For, in the first place, in this kind of death, the heart continues to beat for some time after the cessation of the animal life, and consequently for some time after that of the *action* of the brain."

We now arrive at the inquiry into the influence of the death of the heart over that of the lungs. This inquiry goes no further than to show that, if the heart does not send the black blood to the lungs, that blood will not be oxygenated, and then the lungs, as well as every other part, will die.

As little do we learn from what is contained in the succeeding chapter "On the influence of the death of the heart over that of all the organs," excepting one remark, to which we may hereafter return, namely, "that in reptiles and animals whose pulmonary structure is different from that of quadrupeds, the reciprocal connection between the heart and the brain is not so close, insomuch that the heart, detached from every other part, will continue to beat."

The fifth chapter is "on the influence of the death of the heart, as to the production of general death." Of this we shall transcribe the two leading paragraphs.

"Whenever the heart ceases to act, general death is produced in the following manner:—1st. For want of excitement, the cerebral actions are annihilated, and consequently an end is immediately put to all sensation, locomotion, and utterance. Besides, for want of excitement on the part of the blood, the organs of these functions would cease to act, even supposing that the brain were to remain intact, and exert upon them its accustomed influence. Thus the whole of the animal life is suddenly suspended, and at the instant of the death of the heart, the individual is dead for what surrounds him.

"The interruption of the organic life, which has commenced by the death of the heart, is produced at the same time by that of the lungs. The brain being dead, the mechanical functions of the lungs must cease: the chemical functions of the lungs must cease also, for want of the materials on which they are exerted: the latter are directly interrupted, the former through the medium of the brain."

Our readers will recollect, in our extract from Sir Everard Home's paper in the Philosophical Transactions, that he very justly observes, the heart may cease to beat for some time without the cessation of the powers of the brain; of which Mr. Hunter's case is a well-known instance. But we must agree with Bichat that if pressure is necessary for the action of the brain, it is impossible to conceive how this pressure can be preserved after the heart has ceased to act. It is therefore probable that neither pressure nor motion of the vessels are absolutely necessary for the functions of the brain, though without doubt they cannot be long continued without a regular supply of blood. The rest of the chapter only goes to prove, what no one will doubt, that if the blood is not regularly transmitted, the whole animal must die. We must, however, protest against the following as an universal aphorism:

"It is in this manner (says M. Bichat) also that we die from sudden affections of the mind. The news of a very joyful or a very melancholy event, the sight of a fearful object, of a detested enemy, of a successful rival, are all of them causes capable of producing death. Now, in all these instances, it is the heart which is the first to die, the heart whose death successively produces that of all the other organs, the heart on which the passion is exerted."

In cases of pure syncope, the author is correct. If continued too long, the effect is as he describes; but, where the passion excited has been peculiarly violent, the action of the heart is not merely suspended, but the organ itself instantly dies, and all the other parts of the body die at the same time. This is proved by the immediate change which takes place over the whole body, by the muscles never stiffening, the blood never coagulating, and by the suddenness with which putrefaction follows. We shall not, therefore, enter into the difference of opinion between our author and the late Professor Cullen. Passions of the mind, when violent, seem to produce their effects on every part, and it is only because the effect on the heart is the most obvious to our senses, that it is soonest noticed; but if the effect is less sudden, and long continued, the brain more commonly suffers than the heart: madness ensues, under which the heart is only affected by sympathy, in proportion to the violence of the distemper in the brain.

The sixth chapter is very long, and somewhat diffuse—"On the influence of the death of the lungs over that of the heart." The principal object is to show that the heart does not cease to act merely from the want of oxygenated or red blood flowing into the right auricle, and thus proving a stimulus to its motion. This has been shown so repeatedly in Mr. Hunter's experiments on artificial breathing, that all contained on the subject here might have been omitted. M. Bichat's opinion is, that the death of the heart, and, indeed, of every other part, arises from the capillaries of the arteries being filled with black blood. We should rather say that this is the necessary consequence of death, induced by suffocation; but this is saying no more than that black or unoxygenated blood is unfit for supporting life, which is universally known. Our author goes further, and asserts that such blood is unfit for the purposes of secretion. Of the contrary of this, however, the secretory part of the liver is a standing proof. Mr. Hunter has also remarked that redness is no proof of vitality in blood, as that colour may be imparted to it in almost any stage by the admission of oxygen in any form, even in its neutralised state of nitrated potash. What is much more to the purpose, he has likewise remarked that the dark modena colour is not a sufficient mark that the blood has lost its vital principle, or its properties necessary for secretion, and even for supporting the life life and heat of a part. As a proof of this, he remarks, that whatever produces a stagnation of the blood in the living body, or even a very slow motion, will be sufficient to alter its colour, but not its fitness for all the purposes of a vital fluid. (See Treatise on the Blood, pages 66, 67, and seq.) These remarks render it unnecessary to take any other notice of this chapter than to regret that the author had not made himself master of Mr. Hunter's experiments, and the legitimate result of them. It may be said that M. Bichat has his experiments also. This is very just. But, though they prove all that he wishes, namely, that the heart does not die; or, as he ought rather to have said, does not necessarily cease to act by the admission of black blood into the left ventricle; yet this proves nothing as to the properties of the blood, so far as connected with its colour.

We are now brought to another highly important enquiry, termed by the author "The influence of the death of the lungs over that of the brain." Presuming, by the arguments of the last chapter, that the death of the heart arises, (or, he here more properly writes, that the movements of the heart are paralysed,) because the fleshy fibres are penetrated with venous blood, he proceeds to show that the same may be referred to the action of the brain. The experiments on this occasion are much more decisive, which we cannot wonder at, because the functions of the brain, when suspended by violence, are not so easily restored. The author injected venous blood from the jugular vein of one animal into the carotids of another, and found the consequence always mortal. The brain, to use his language, died first, consequently the animal life was soon extinguished, and the death of the organic soon followed. These experiments appear to have been all of them judiciously conducted. To satisfy himself that it was not merely the absence of red blood which produced this effect, water was injected, and produced no permanent ill consequences. One elegant experiment was the injection of venous blood oxygenated after it was taken from the body; but the effect was the same as in common black blood. Another experiment is not less worth noticing, as it shows the wish of the author to meet every possible objection, and to vary his proofs in every form.

"From the influence (says he) of the black blood over the heart, the brain, and the rest of the organs, it was my opinion, that persons affected with varicose aneurisms would perish less quickly from asphyxia than others, because the red blood passes into the veins, and traverses the lungs without requiring alteration. Accordingly, it should be capable of keeping up the cerebral action.

"To be assured if this suspicion were well founded, I made a communication between the carotid artery and jugular vein of a dog, by means of a curved tube. The pulsation of the artery was thus communicated to the vein. I afterwards asphyxiated the animal by stopping the trachea; but the phenomena of death were little different from those of common asphyxia.

"We may conclude with certainty, from the various considerations and experiments exposed in the present chapter,

1st. That, where the chemical phenomena of the lungs are interrupted, the black blood acts upon the brain, as it does upon the heart, by penetrating the tissue of that organ, and depriving it of the excitement which is necessary to its action.

"2dly. That its influence is much more rapid upon the first than on the second of these organs.

"3dly. That it is the inequality of such influence which occasions the difference in the cessation of the two lives in the case of asphyxia. The animal life is always annihilated before the organic life.

"We may conceive, from what has been said in this and the preceding chapter, how unfounded are the suspicions of those who have supposed that the brain, after the separation of the head from the body by the guillotine, might live awhile and have sensation. The action of this organ is immediately connected with its double excitements—1st, by motion; 2d, by the nature of the blood which it receives. Now, when the interruption of such excitement is sudden, the interruption of every kind of feeling must also be sudden.

"When the chemical functions of the lungs are suspended, the disturbance induced in the functions of the brain has indeed a very considerable influence on the death of the other organs; nevertheless, such disturbance is the beginning of death only in the animal life, and even then is connected with other causes. The organic life ceases from the sole presence of the black blood among the different organs. The death of the brain is only an isolated and partial phenomenon of asphyxia, which does not take place in any particular organ, but in all alike. We shall explain this assertion in the following chapter."

In the eighth chapter M. Bichat proceeds to consider the influence of death in the lungs over that of the organs in general. Preparatory to this, we have a section to show the phenomena attending the production of black blood when the chemical functions of the lungs are suspended. This commences with a very just observation concerning the uncertainty of such experiments on frogs and other amphibious animals, on account of the slowness of the respiration, and the comparatively small quantity of blood which passes through their lungs. His experiments are, therefore, very properly confined to animals with a double heart, and a pulmonary system similar to the human. In these he regrets that for the most part the vessels are less transparent, which considerably increases the difficulty of such examinations as require a precise knowledge of the blood's colour. The plan pursued was similar to what he has represented before, namely, to fix a tube with a stop-cock in the trachea. By these means the life of the animal may be protracted for some time, respiration being interrupted or permitted according to the intention of the experimenter: add to this, various kinds of air may be admitted at different times, and the effects accurately traced. Another part of the experiment consisted in opening a large artery. A small one, it was observed, was not equal to showing the early changes in the colour of the blood, on account of the slow circulation in that series of vessels. To prevent the too sudden flow of blood, the carotid was opened, and a small stop-cock attached to it. Having prepared a dog in this manner, the result of all his experiments were as follows:—1st, that the length of the interval during which the blood retains its red colour, is in direct proportion to the quantity of air contained in the lungs; 2dly, that as long as there remains any quantity, however small, of respirable air in the cells of the lungs, the blood will preserve more or less of its crimson colour; 3dly, that this colour diminishes in proportion as the respirable air diminishes; and 4thly, that the blood is exactly similar to that of the veins, as soon as the whole of the vital air in the extremities of the bronchiæ has been exhausted.

During these examinations, a curious phenomenon struck the author, namely, that, when the stop-cock was shut, if the animal had an opportunity of moving his body so as to agitate his chest, the colour of the blood was longer in becoming black. This he very justly imputes to the circulation of the minutest particles of air in the chest, caused by this agitation; and the consequence of which is, that every such particle of air is taken into the blood. But the most interesting circumstance in the above detail appears to us that it accounts for the perpetual motion, and even attempt to press the chest, which we see in those who are fearful of being suffocated by any obstruction in the trachea. It seems also to show the propriety of a common practice, that of beating against the back of a person under such circumstances; thus exciting a contraction of all the muscles of respiration, as well as those of the abdomen, which must further conduce to this circulation of what air is left in the lungs.

The next experiment, which the former naturally led to, was was an examination of the phenomena of the blood, when the stop-cock was opened, and atmospheric air admitted, after the blood had become black. In all these experiments the author found that he never could restore motion to the heart after it had once ceased from the black blood penetrating its substance.

The author next tried the effect of different kinds of air admitted to the lungs. To accomplish this, he fitted bladders to his tube, and filled them alternately with hydrogen and carbonic acid gas. Under these circumstances it was found that the animal lived longer than when deprived of every means of breathing, which is imputed to his greater capacity of agitating his lungs, and probably few gasses are entirely free from oxygen or atmospheric air. When the bladder was filled with pure oxygen, the blood did not assume a higher colour, but the animal lived longer, the blood becoming black more slowly.

Some experiments follow to show that the black blood will continue to circulate for some time through both series of vessels. This is proved in a variety of ways, but a single observation of the author's shows how little necessity there was for many of his unfeeling experiments. "There are," says he, "few parts where the influx of the black blood is more visible than in the skin: the livid spots, so frequent in dyspnœa, are only the effects of the obstacle it meets in its passage towards the general capillary system, to the organic contractility of which it is not a sufficient excitant." Whatever may be the cause, the effect is certain, that the black blood does arrive at the superficial arteries, and that the cheeks and lips grow first purple and then black, and so continue as long as the blood is not oxygenated through the medium of the lungs. The following observations contain some curious facts, which may lead to conjectures that in some animals, if not in all, the fœtus is fed with venous blood.

"I was desirous of making use of the power which we possess of changing the colour of the blood, for getting some insight into the influence of the circulation of the mother upon that of the fœtus: accordingly, I procured a bitch big with young, and asphyxiated her, by closing a tube adapted to the trachea. About four minutes after she had ceased to breathe, I opened her: the circulation was going on. I then cut into the matrix, and exposed the cord of two or three of the fœtuses. The artery and the vein were both of them full alike of venous blood.

"Had I been able to procure other bitches in a similar state, I should have repeated this experiment in another manner. I should, in the first place, have compared the natural colour of the vein vein with that of the artery. In many of the young of the guinea-pig, the difference appeared to be much less than it is in the adult animal. In many circumstances, indeed, I could perceive no difference whatever. Both the arterial and venous blood were equally black, though the respiration of the mother was in no wise impeded by the opening of the belly. Secondly, I should have closed the tube in the trachea, and then have observed whether the change in colour of the umbilical artery of the fœtus (supposing the blood of the artery to be different from that of the vein) were correspondent with that which would inevitably take place in the blood of the mother. Experiments made with a view to these circumstances, and on large animals, might probably throw much light upon the mode of communication between the mother and the fœtus. Observations are also much to be desired with respect to the colour of the blood in the human fœtus, and the cause of its passage from a livid colour to the very marked red which it assumes some little time after birth.

"I might add a number of examples to these which I have already related of the blackening of the organs by the venous blood. Thus, the kidney of a dog exposed, while the animal is dying of asphyxia, is much more livid than in its natural state; the spleen also and the liver, when divided, emit only black blood, instead of that mixture of red and black blood which is observable, in the section of these organs, upon an animal which breathes freely.

"But I trust that we have facts enough to establish it as a certainty, 1st, that the black blood, after the interruption of the chemical functions of the lungs, continues for some time to circulate; and 2dly, that it penetrates into the organs, where it replaces the red blood. These circumstances explain the reason why, on opening the body, we always meet with black blood, even in the vessels which are destined for the circulation of arterial blood.

"In the last moments of existence, of whatever death the individual may have died, we shall always observe the lungs become embarrassed and cease to perform their office, for some time previous to the total suspension of the functions of the heart. The blood makes its circle through the system, after ceasing to receive the influence of the air, and consequently in its venous state: accordingly it must remain so in the organ in every case, although the circulation be much less evident than in asphyxia, for it is in this circumstance that consists the great peculiarity of asphyxia. The following phenomena may now be easily understood.

"1st. When the left auricle and ventricle, together with the large divisions of the aorta, on opening the body, are found to contain blood, such blood is always black. The fact is familiar to those who are in the habit of dissecting. In exercising my pupils on the surgical operations, I have always observed, that when the open arteries are not entirely empty, their contents are composed of venous blood.

 "2dly.

"2dly. The corpus cavernosum is always gorged with this sort of fluid, whether flaccid, or in a state of erection; for I have seen it in the latter state in two subjects brought to my amphitheatre. One of these men had hanged himself; the other had died of concussion of the brain.

"3dly. The blood which is found in the spleen is never red; but, sometimes on the exterior, and sometimes on the concave, surface of this organ, I have observed spots of a scarlet colour, for which I cannot account.

"4thly. After death, the mucous membranes lose the red colour by which they are characterized during life. They assume a black and livid hue.

"5thly. Blood extravasated in the brain of persons in a state of apoplexy, is almost always found to be black.

"6thly. Sometimes, instead of accumulating inwardly, the blood injects the surface of the body. In such cases, the face, the neck, and shoulders swell, and are infiltrated with blood. I have frequently remarked this sort of phenomenon in the subject, but have never found it coincide with any internal extravasation. The colour of the skin is then of a purple or deep brown, an evident sign of the sort of blood with which it is injected; and is evidently produced by the stagnation of the black blood in the external capillary system, not by the reflux of blood from the veins."

We cannot help remarking, that every thing important in the latter part of these extracts has been already much better observed by Mr. Hunter, (see his Treatise on the Blood, &c.) who, by a variety of experiments, proved that blood stagnating in an artery, or in any part of the living body, always became black. In the instance above noticed by Bichat, the progress is simple enough. By the mode of death the arteries have been unable to contract, and the blood has remained in them: the same cause has prevented the coagulation of the blood in the heart and larger arteries, and, probably had it been noticed, no stiffening of the body ever took place. All this has been most accurately noticed by our English physiologist, and explained in a most satisfactory manner in his remarks on the recovery of persons apparently drowned.

The chapter before us concludes with some general experiments to prove that the black blood will not support the functions of life. This is already so well understood, that we shall only extract a single passage, which we recommend to the careful attention of some younger experimenters in our own island.

"In general I have had occasion to remark, that during the circulation of the black blood in the arteries, no fluids appear to issue from the different secreting tubes. But I confess that in all these experiments, and in other similar ones which I have made, the the animal is too much agitated, and the limits of the experiments too circumscribed, for any thing like a well-founded judgment to be formed on the subject in question. It is chiefly from analogy, then, that I am led to conclude, that the black blood is unfit for the purposes of exhalation and nutrition: such supposition also accords well with divers of the phenomena of asphyxia. 1st. The want of exhalation from the skin during the state of asphyxia, is probably the reason of the phenomena of the animal heat in such sort of death. 2dly. In asphyxiating animals very slowly during digestion, I have uniformly observed, that the bile ducts and duodenum contain a much less quantity of bile than they do at such time when these parts are exposed in the living animal. 3dly. As the blood loses nothing from the exercise of these functions, it must, of course, accumulate in the vessels; and, in fact, it is very fatiguing and unsatisfactory to dissect the bodies of those who have been hanged, or asphyxiated with the vapours of charcoal, from the fluidity and abundance of their blood. But this abundance, perhaps, may depend upon the weakness of the absorbents. In other sorts of death, the absorbents continue for some time to act upon the serous portion of the blood remaining in the vessels. In asphyxia there is neither secretion nor absorption."

This is, at least, a candid confession of the uncertainty which must attend every experiment on living animals. We pass over the succeeding chapter on the influence of the death of the lungs over the general death of the whole body, as containing little more than what is to be met with above.

Some remarks follow on the influence of the death of the brain over that of the lungs. Here we meet with much of what has lately been exhibited in England, and in both cases to very little account. From experiments in which so much violence is done as must be required in order to tie or cut through the cardiac nerve, what conjectures can we form concerning the influence of the nervous system abstracted by reasoning from the mere divesting a part of the nervous influence. The author's conclusions, however, are as candid as can be expected. He shews that the lungs do not cease to act by the direct influence of the cessation of action in the brain. But this is only proved by shewing that the influence of the nerves subservient to the muscles of respiration are not necessary for the continuance of breathing. Nervous influence is extremely uncertain, and sympathies are scarcely less various than anastomosing branches of blood vessels. These experiments, however, will always be interesting; they are good for as much as they prove, and it redounds to the author's credit, that on many occasions he is so cautious in drawing conclusions from them. All he infers is, that "the death of the lungs is occasioned *indirectly* by the death of the brain." It is painful to be obliged to object to the language of philosophers, but, without greater accuracy in our expressions, to what purpose is all our reasoning. The death of the brain cannot take place without the death of every other part, and our author has only been talking about the cessation of communication. When the head or brain is entirely separated, the other parts die slowly, but not instantly. Need we require a stronger proof of this than the experiments of artificial breathing, so nicely conducted by Mr. Hunter, and since repeated by Mr. Brodie. All these remarks may be extended to the subsequent chapter, "on the influence of the death of the brain over that of the heart;" and the two succeeding chapters, "on the effect of the death of the brain on the other organs and over every part of the body." This last chapter we shall transcribe, because it is short, and because it offers a most lively instance of the importance of distinguishing between death and the cessation of action; the want of which distinction we have so often remarked, and may now conclude with asserting, that it renders the whole work useless excepting as a series of well conducted and faithfully related experiments; which the disciples of a more correct physiologist may turn to good account in their various inquiries.

"*Of the Influence of the Death of the Brain over that of the Body in general.*

"From the consideration of what has been said in the preceding chapter, nothing can be more easy than to form an accurate idea of the manner in which the phenomena of general death, commencing by the brain, are concatenated. The series is as follows:

"1st, The cerebral action is annihilated. 2dly, There is a sudden cessation of sensation and voluntary motion. 3dly, A simultaneous paralysis of the intercostals and diaphragm. 4thly, An interruption of the mechanical phenomena of respiration and the voice. 5thly, An annihilation of the chemical phenomena of the lungs. 6thly, A passage of black blood into the arteries. 7thly, A slowness of circulation owing to the influx of such blood into the arteries, and the absolute immobility of all the parts, of the intercostals and diaphragm in particular. 8thly, The heart dies and the general circulation ceases. 9thly, The organic life vanishes. 10thly, The animal heat, which is the product of all the functions, disappears. 11thly, The white organs die.

"Though in this kind of death, as well as in the two preceding kinds, the functions are suddenly annihilated; the parts retain, for a certain time, a number of the properties of life. The organic sensibility and contractility, continue for some time, to be manifest in the muscles of the two lives; and in those of the animal mal life, the susceptibility of being affected by the galvanic fluid is very satisfactorily marked in the muscles of the animal life.

"This permanence of the organic properties, is nearly the same in every case; the only cause which affects it, is the slowness with which the phenomena of death have succeeded each other. In every case where their duration has been the same, whatever may have been the cause of death, experiments instituted upon these properties, are attended with similar results; for it is evident that concussion of the brain, luxation of the vertebræ, the section of the spinal marrow, apoplexy, compression of the brain, or inflammation, are all of them causes which are attended with a like effect.

"The same, however, is not the case with respect to the asphyxiæ produced by the different gasses. We have shewn the reason of this in the more or less deleterious nature of the gasses which produce asphyxiæ.

"The state of the lungs also, is very various in the bodies of persons who have died from lesions of the brain. This organ is sometimes gorged and sometimes almost empty: it shews, however, whether the death of the individual has been sudden or gradual. The same indication may be had from the state of the exterior surfaces.

"The death, which is the consequence of disease, commences much more rarely in the brain, than in the lungs. Nevertheless, in certain paroxysms of acute fever, the blood is violently carried to the head, and is the occasion of death. The concatenation of its phenomena are then the same as take place in sudden death.

"There are a great number of other cases besides those of fever, where the commencement of death may be in the brain, though the brain itself may not have been previously affected by the disease. In these cases, the state of the lungs is very various; but little can be learnt from it with respect to the nature of the disease. It is only an indication of the manner in which the functions have been terminated."

By the third paragraph of this chapter, it evidently appears how much the author was embarrassed for want of a due distinction between a total cessation of action and absolute universal death. What can be more absurd than to talk of *dead parts* retaining the *properties of life!*

An additional note is added, containing M. Bichat's mode of considering the great sympathetic nerve. This we shall transcribe, as an easy illustration of the connection between the neurology of the past and present century.

"This nerve, says he, is considered as a medullary cord, extending from the head to the sacrum;—in this course it is represented by anatomists, as sending different ramifications to the neck, breast, and abdomen, as having a distribution analogous to that of the nerves of the spine, and as deriving its origin, either from these nerves, or those of the brain. Whatever be the name by which it is designed, sympathetic, intercostal, or trisplanchnic, the manner of regarding it is invariably the same.

"But there exists no such nerve as that which is described under these names. That which is taken for a nerve, is nothing more than a series of communications between different nervous centers placed at different distances from each other.

"These nervous centers are the ganglions. Disseminated in different regions, they have all of them an independent and isolated action. They are, each of them a focus, which sends out in different directions a number of ramifications, which in the several organs to which they are distributed, are the conductors of the irradiations of the focus from whence they escape.—Of these ramifications, there are some which go from one ganglion to another, and as these branches, which unite the ganglions, compose by their aggregate a sort of continuous cord, such cord has been considered as an isolated nerve, but it is no such thing; these branches are nothing more than communications, simple anastomoses, and not a nerve.

"This will be evident, when it is considered that the communications are frequently interrupted. There are subjects, for example, where a very distinct interspace is found between the pectoral and lumbar portions of that, which is called the great sympathetic; it seems to be divided at this spot. Every anatomist must have remarked, that sometimes a single branch, and sometimes many, pass from one ganglion to another, and this particuliarly between the last cervical and first dorsal ganglion. Besides, the volume of these branches is singularly various, and, after giving out a number of branches, the sympathetic is larger than ever.

"These different considerations are a manifest proof that the communicating branches of the ganglions should no more be considered as a continued nerve, than the branches, which pass from each of the cervical, lumbar, and sacral nerves, to those which are immediately above and below them. In fact, notwithstanding these communications, each pair of the latter mentioned nerves is regarded as a separate pair.

"In like manner each ganglion should be considered separately, and the branches be described which proceed from it.

"For this reason, continues Bichat, in my descriptions in future, I shall divide the nerves into two great systems; the one emanating from the brain; the other from the ganglions, &c. The first has a single center, the second a number of centers. This manner of considering them, will present them such as they actually are in nature.

What anatomists, for instance, can there be found, who has not been struck with the difference which exists between the nerves of these two systems. Those of the brain are larger, but less numerous;—are whiter, denser, and less subject to variations than the others. On the contrary, the general character of the nerves of the system of the ganglions consists in extreme tenuity, great number, a greyish colour, softness of texture, and great variability.

"Besides,

"Besides, this division of the general system of the nerves into two secondary systems, agrees exactly with my division of life. The external functions, sensation, locomotion and the voice, depend on the cerebral system. On the contrary, the greater number of the organs of the internal functions have their nerves, and consequently their principle of action from the ganglions. From the former are derived, the animal sensibility and contractility; where the latter only are found, there are only to be found the organic sensibility and contractility.

"I have said elsewhere, that the term of this species of sensibility, and that the origin of the corresponding contractility, reside in the organ in which they are observed; but perhaps this term and origin are to be found at a greater distance; perhaps they exist in the ganglion from which the organ receives its nerves, just in the same manner as the brain is the term and origin of the animal sensibility and contractility. If this be the fact, as the ganglions are very numerous, we may easily conceive why the powers of the organic life have not a common center.

"From all this, it is manifest, that there exists no great sympathetic nerve, &c. &c."

All the sympathetic properties of this nerve are well described by Winslow. The distinction made by Bichat between a real nerve, and such a substance as is here described, though not entirely new, is well expressed; and, whatever may be the imperfections of the work, it certainly shews genius, industry, and integrity. We may add, that, considering the whole was conducted in a part of the world where Mr. Hunter is so little known, the author is fairly entitled to all the reputation he has acquired.

---

*A General Dispensatory, or Arrangement of the Pharmacopœias of London, Edinburgh, and Dublin; in which the Strength of various Preparations is expressed by Pharmaceutical Numbers; the different Synonyms of each Article, Doses, Qualities, Chemical Numbers, &c. are likewise added: and to the whole are prefixed, some Observations upon the present State of the Nomenclature of Pharmacy.* By S. Rootsey, F.L.S. Bristol, printed for Baldwin, Cradock, and Co. London. pp. 142.

This is one of the most extraordinary little books we ever remember to have met with in the progress of our labours. In the space of about one hundred and fifty pages, more than half of which are devoted to *meteria medica* and index, the author has actually accomplished all that he promises in his long title-page. After this observation, our readers will not expect us to offer an analysis of what is expressed with as much brevity as possible, and perhaps even in fewer words on some occasions than could be wished. The title itself too shews the intention of every division. We must, therefore, content ourselves with short extracts, to shew the manner in which so much is compressed into so short a compass. The preface will assist us a little, and, as it is short, we shall transcribe the whole.

"In writing the following work, my principal object was to explain my method of expressing the composition and the strength of various medicinal preparations by means of pharmaceutical numbers; and having been for some time in the constant use of what I conceived to be a more philosophical language of pharmacy, I determined upon uniting both of these ideas in one publication, and printing them in the form of a dispensatory as they now appear.

"The former presents us advantages so important and so obvious, that I consider no apology can be necessary for my making it known. But, as my own opinion of the necessity of nomenclatural reform may not with my cotemporaries possess any weight, I shall adduce the opinions of some of those philosophers who are justly considered as high authority.

"I have been stimulated in my undertaking by the advice which the illustrious Bergman once gave to M. de Morveau, 'spare no improper names, those who are learned will always be learned, and those who are ignorant will thus learn sooner.' And I may also urge as relevant to this point the arguments of the unfortunate Lavoisier, whose zeal and freedom in the promotion of science have never been surpassed. 'As ideas,' he justly observes, 'are preserved and communicated by means of words, it necessarily follows that we cannot improve the language of any science without at the same time improving the science itself; neither can we on the other hand improve a science without improving the language or nomenclature which belongs to it.'

"Sir H. Davy, who has devoted much of his attention to this subject, likewise judiciously remarks (p. 46 of his Elem ) that 'a theoretical nomenclature is liable to continued alterations; *oxygenated muriatic acid* is as improper a name as *dephlogisticated marine acid;* every school believes itself to be right, and if every school assumes to itself the liberty of altering the names of chemical substances in consequence of new ideas of their composition and decomposition there can be no permanency in the language of the science, it must always be confused and uncertain.' And in his advertisement he says, ''till a more simple system is adopted, innovation will be censured, sometimes perhaps even when it is necessary, and neology generally brought forward as a reproach.'

"But still more weighty and impartial may appear to some, the arguments of Bacon and Locke, who flourished at an earlier period, and whose luminous writings have served, and should still serve, as a compass to direct our course in the promotion of philosophy. To those who possess the philosophic spirit of the former, or who are acquainted with his works, it will be unnecessary for me here to to adduce what he has said 'de Idolis Fori.' And the latter has given us in our vernacular tongue several excellent rules as criteria of the imperfection of any particular language (of pharmacy for instance), which are so much to the present purpose, that I cannot forbear to conclude with the following transcript from his essay upon the Human Understanding.

" 'The ends of language,' he remarks, 'being chiefly these three—1, to make known one man's thoughts or ideas to another; 2, to do it with as much ease and quickness as possible; and 3, to convey the knowledge of things;—language is either abused or deficient when it fails of any of these three.' After treating at some length upon certain abuses, he thus proceeds:

" 'To remedy the defects of speech before-mentioned in some degree, and to prevent the inconveniences that follow from them, I imagine the observation of these following rules may be of use. 1. A man should take care to use no word without a signification, no name without an idea for which he makes it stand. 2. The ideas he annexes to his words, if they be simple, must be clear and distinct; and, if complex, determinate. 3. Care must be taken to apply words as near as may be to such ideas as common use has annexed them. 4. Because men, in the improvement of their knowledge, come to have ideas different from the vulgar and ordinary received ones, for which they must either make new words, or else use old ones in a new signification; therefore it is sometimes necessary, for the ascertaining the signification of words, to declare their meaning, where either common use has left it uncertain and loose, or where the term, being material, is liable to doubtfulness or mistake, which may be done by synonyms, by exhibition, by definitions, by drawings, and by adhering to one signification.' These remedies, he continues, are necessary to the improvement of philosophy; and 'though the market and exchange must be left to their own ways of talking, and gossipings must not be robbed of their ancient privilege; though the schools and men of argument would, perhaps, take it amiss to have any thing offered to abate the length or lessen the number of their disputes; yet those who pretend seriously to search after or maintain truth, should think themselves obliged to study how they might deliver themselves without doubtfulness or equivocation.' 'For he that uses words without any clear and steady meaning, only leads himself and others into errors; and, if he does it designedly, he ought to be looked on as an enemy to truth and knowledge.' "

Perhaps the author's having hit upon our favourite topic, and even concluded with a strong expression in favour of accurate language, may have induced for him a sympathy which may betray us into some partiality. This we trust, however, will be a venial offence. The following are his remarks on nomenclature, and his rules of etymology.

" LAWS OF NOMENCLATURE, which no author is at liberty to supersede, supersede, and which it is the duty of critics to see enforced. These laws are scattered over the writings of various authors upon language, and I have endeavoured to collect those which were necessary to my purpose. Linnæus, in his different works, has laid down many rules, so excellent, that as soon as they were promulgated they were generally embraced, and the impulse which was given to natural history by his writings will make his name eternally dear to its votaries. In the nomenclature of inorganic substances, the proposals of the French chemists, Lavoisier and others, have met with great approbation. But the discoveries which have been the consequence of nomenclatural reform, have rendered some changes necessary.

"The language of science, unfortunately, is not calculated to be the language of business; and from the length of the names of organic substances, and uncertainty of those of inorganic, neither have been generally adopted in pharmacy, nor has any one succeeded in giving commercial names that have been universally approved of. Notwithstanding this, much has been written upon the subject, but it has commonly been to point out a few errors, and not to establish the whole upon a grand and lasting foundation. Finding, in the nomenclature of the three British pharmacopœias, important differences in many names, such as chamomile, blistering fly, &c. I found myself obligated to give my reasons for selecting those which I have preferred; and the manner in which I have executed this task will, I hope, exempt me from censure, considering that it is the duty of authors to use those names which they feel to be the most proper and the most classical. In doing this I have proceeded with caution, and have arranged the names of species according to my view of their classicality and propriety, by which it will appear, that my proposed alterations extend only to names indubitably aukward, uncommercial, or unclassical. It is certainly desirable that the language of pharmacy should be classical; and there are those who, 'in defiance of all undue authority,' will ever oppose the depravity and barbarism into which we are at present plunged, and which must ultimately be extirpated, so that the taste of the last age will be succeeded by another less corrupt.

"Law I.—*When a name is once attached to a single species, it must not be given to another, except as an epithet.* In other words, *No name ought to be rendered equivocal, that is not so already.*

"Law II.—*When a name, usually considered as generic, is used specially, it must be understood as implying the original species,* if it can be satisfactorily ascertained, and if not contrary to the generally-received opinion. It will also be necessary for the author who uses it in that sense to give some advertisement of it.

"Law III.—*When a new species is introduced into commerce, it must have a new name of one word as pure as the Linnæan genera.*

"Law

"LAW IV.—*Names must be as classical as possible*, and it is the duty of an author to choose and to adopt from amongst the synonyms of a species the best name with which he is acquainted."

"RULES OF ETYMOLOGY.—1. The character by which a species is distinguished, such as its resemblance to something else, its quality or property, its colour, its use, or its native situation when constant, suggest the most appropriate names, as *Pterocarpus*, *Chenopodium*, *Dulcamara*, *Glycirrhiza*, *Erythrodanum*, *Pyrophorus*, *Origanum*, *Cydonia*.

"2. New names must always be of the second quality if possible, but never so bad as the sixth. If of the first quality, they are destitute of euphony.

"3. Modern writers have taken the liberty of naming species from their discoverers, as *Spigelia and Quassia*, but such as *Geoffroya*, *Witherites*, *Swietenia*, *Kraschenninikofia*, &c. are, in my opinion, rather too incongruous.

"4. Names in point of orthography and etymology must not be contrary to analogy, as Barytes for Barites, Ipecacuanha for Ipecacuania, &c.

"5. Names must not be too long nor inappropriate, as Hypophyllocarpodendron, Spermaceti, Pulvis cretæ compositus cum opio, for Opium cretaceum compositum.

"6. The solution of an inorganic or chemical substance, including oils, essences, soaps, camphor, and the like, in a menstruum, may either receive a name from both, that of the solvend being in the genitive case, as Aqua calcis, lime-water; or the solvend may give an epithet to the menstruum, for salt water or solution of salt is rendered into Latin by Aqua salina probably better than by Aqua salis.

"7. If we suppose that the common names of salts are of this description, the acids may very properly be called Nitrate, Oxalate, &c.

"8. All organic preparations are to be named in the same manner as inorganic, except Decoctions, Infusions, Liquors, Elixirs, and Tinctures.

"9. In Pharmacy, water impregnated with any air or gas may take the name of that air or gas, as Murias, &c.

"10. When two or more articles are dissolved in a menstruum, the basis must be in the genitive case, and the other must give an epithet, as Infusum sennæ tartarizatum, Tinctura cinchonæ composita. And, when a name contains no epithet, it must be considered as simple: for instance, Tinctura cardamomi, &c."

A section on Definition follows, replete with method and accuracy; some remarks on *Icons*, referring to the descriptions, and where they are to be met with, the plates of writers on natural history; Synonymy.

The second chapter is inscribed Statics, and comprehends a correct and classical view of weights and measures. This is particularly useful, on account of the facility and brevity with which the three Pharmacopœias are connected. Either, however, we have overlooked the passage, or the author has omitted the thermometrical degrees in speaking of the cubical measures of liquids. This may be thought of less consequence, as all the parts must be similar to the whole; but still there seems to us the want of a standard.

Classification next attracts our attention. The two first divisions are zoology and botany; the third chemistry. In the progress of the work, the author shows ample cause for this last division, and the various subdivisions into which it is formed. In zoology, he recommends Turton and Bingley as the most convenient, though not complete, writers on those subjects. In botany, Sir J. E. Smith and Galpine. We think he might have stopt here; but in the space of two or three pages, he attempts an analysis of the Linnæan system. This is enough to show how familiar the author is with the subject, but can hardly be expected to satisfy a tyro. Chemistry comprehends chemical numbers,—a very ingenious and not less perspicuous little abstract of which is given; chemical attraction, with an illustrative table; specific gravities [in this place the author has fixed the thermometric point]; pharmaceutic numbers, which, being connected with the index, we shall transcribe.

"In considering the proportion which the solvend bears to the menstruum, I constantly take the solvend for unity, and the number for the menstruum I affix to the preparation. Thus, in preparing Tincture of Squills, 4 ounces are added to a quart, or 32 ounces of proof spirit, which being 8 times as much, I annex the number 8 to that preparation. Again, the Edinburgh College direct Unguentum Cerussæ to be prepared by mixing one part ceruse with 5 of simple ointment, the number attached to that article must be therefore 5. Thus it is evident that this plan, while it supersedes simple formulas, answer the purposes of the table appended to the Pharmacopœias. For, if we turn to opium, we shall find one grain united to as many grains of other substances as the number indicates.

"This invention may be applied to the construction of a

SYNOPTICAL PHARMACOPŒIA, which, from its smallness, may very conveniently be suspended in a library, dispensary, or shop; for which purpose, two copies will be given with each one of this work. This table will very much assist the memory in retaining the composition of medicines, and I have myself found it of very extensive service for some time past."

This division closes with a very useful contrivance for an easy reference to doses; and the chapter concludes with an explanation of the signs by which the authorities are marked in the different indexes.

The

The last chapter is on manipulation, and contains an useful little compendium. The materia medica follows, first alphabetically arranged, and afterwards remedially, in the manner of Dr. Cullen. The first of these is very copious, and requires a great deal of attention before it can be well understood. It is indeed connected with almost every other part of the book, both preceding and following it. When the reader has made himself master of it, he will find it extremely convenient on every occasion on which he wishes for information.

A list follows of those articles of materia medica which are usually imported, and of the places from which they are brought. Lastly a copious index and a synoptic pharmacopœia.

Such are the contents of a work new in its kind, which must have cost the writer infinite trouble; and for which we, in common with the rest of his readers, are ready to acknowledge our obligations.

---

*Edinburgh Medical and Surgical Journal*, No. XLV. for January, 1816.

Art. I.—*Case of Carotid Aneurism.* By Mr. Thomas Duncan.

This case is extremely interesting, and very candidly related. We cannot blame the author for not attempting an operation which he admits he ought to have performed, and from which he was only withheld by want of courage, and of knowing what had been accomplished in Europe. The event was as might be expected, but earlier than in aneurisms which occur on the extremities. This may be accounted for by the proximity of the heart, and the want of any resistance to the tumour from a neighbouring bone. The case seems to have occurred in the West Indies, but no date is affixed. The subject was a negro.

Art. II.—*Case of singular Malformation of the Heart, with Remarks on Deranged Circulation.* By James Thomson, M.D. Edinburgh.

This case, and the reflections of the author, are important. We shall transcribe the first, and give the substance of the latter, with our own remarks.

"J. R. ætat. 46. The account this patient gave of her complaints was, that till the last summer she had enjoyed tolerably good health, when she remarked her lips and mouth to turn of a dark colour; her health was not materially altered. She remained much in the same state till three months ago. She then began to complain of her breathing, and the blueness became much more

 general; general; but it is only during the last three weeks that she has been forced to keep her bed. During the time I saw her, which was only three days before her death, she had all the appearance of a person labouring under the severest symptoms of hydrothorax. The pulse was not very much altered, rather feeble; she could only endure the half erect posture, and breathed with very great difficulty. She was ordered to be let blood, and lost nearly eight ounces. Felt no relief, and died in sixteen hours after.

"*Dissection.—External appearances.* Not much emaciation; blue colour more faint than during life; the chest sounded ill when struck, especially the left side; and there was a peculiar form of the thorax, very frequent in cases of diseased heart.

"*Thorax.* Pericardium contained six ounces of a clear fluid; the right cavities of the heart enormously distended with black blood; the right auricle thickened and enlarged in a small degree. In the seat of the foramen ovale, there was a circular aperture sufficient to admit the points of *the four fingers* to a considerable extent; the pillars of the foramen ovale were not distinct. The direction of the aperture was not such as could warrant one in saying, that it was more particularly directed from one side or another. The right ventricle was considerably enlarged and thickened; left auricle rather small; left ventricle natural, but, as usual, contracted; great quantity of blood at the commencement of the aorta; the ductus arteriosus was quite impervious. The lungs were adhering in most places, and their substance was dark-coloured, and full of blood and air; the left lobe at the inferior part contained some extravasated blood. The veins were much distended with blood, particularly those of the liver. The trachea at the bifurcation was much reddened; the stomach internally had a red spotted appearance; the inner surface of the intestines was of a very dark colour throughout.

"The case which I have now detailed is interesting, both from the *unusual* appearance presented on dissection, and also from the speculations it gives rise to, as tending to illustrate some obscure parts of the circulation.

"The first question that naturally suggests itself is, whether this was an original malformation, or a change of structure arising at an after period of life? If it proves to be a malformation, we must show how so singular a deviation could have existed for so long a period without inducing more serious derangement in the system of circulation.

"If we view it as an alteration in structure occurring after birth, we must point out the causes and the time of its formation. After considering the subject attentively, the following circumstances have induced me to refer it to an original malformation.

"1st, The appearance of the aperture, the edges of which were rounded and thickened, indicated that it was not of recent formation; as also, that it was not the foramen ovale merely remaining open, for, in that case, there should have been the two pillars as they they are called, and the aperture should have been of an elliptical form, an appearance which it acquires as the child advances nearer to maturity.

"2dly, At birth, and for some time after, a passage must remain in the seat of the foramen ovale, and ductus arteriosus; yet no unusual appearance takes place, unless some cause deranging the circulation occurs, and then, as we shall afterwards endeavour to show, the fœtal circulation is often again in part renewed.

"The instances in which the foramen ovale remains open are so common, that few apply them to explain the cause of any symptom during life; for at all ages the aperture occurs of various sizes, and its being always unlooked for, sufficiently shows the importance we should attach to it.

"3dly, Bichat injected two different kinds of fluids into the auricles at the same time, and found that they did not intermix, though the foramen ovale was open. This celebrated anatomist seems to think that it is impossible for any fluid to pass from the one auricle into the other, however large the aperture may be.

"4thly, In cases where a communication is found between the ventricles, a striking analogy is observed with the one I have just related. Little derangement happens, as long as both ventricles continue to send out the same quantity of blood; but the moment this relation is lost, the dreadful effects are easily distinguished.

"5thly, Cases of an opening in the septum of the auricles are extremely rare. I find one, however, mentioned by Corvisart. It is particularly detailed in Observation 44, page 279, of that elegant work. The patient was 57 years of age before he complained, and that was seemingly induced by repeated accidents. The right auricle was extremely dilated, and an opening of more than an inch in diameter was found in the septum of the auricles.

"These facts may persuade most of the accuracy of the opinion which considers the aperture as an original malformation. It is certainly more consistent with our present knowledge to view it as such, than to refer it to ulceration or rupture, induced by a violent fit of coughing.

"We must, however, prove how this patient enjoyed her health for so long a period, before admitting implicitly the above statement as a correct view of the subject. We shall therefore endeavour to show that part of the fœtal structure remaining in adult life, has little influence in deranging the health, unless some additional cause arises.

"In examining the cases mentioned by authors who treat of diseases of the heart, we find sufficient proof to convince us, that, on dissection, many appearances of altered structure have presented themselves, which, from the state of symptoms during life, we never should have suspected. In these cases, little variation will be found to have taken place in the relative size of the cavities of the heart.

"We

"We have already noticed, that the foramen ovale is found open so frequently, that anatomists look on it as a matter of little consequence. The observation only holds true, however, where the relative size of the auricles remain the same; for the moment the right increases in size or strength, or any other derangement of the lesser circulation is added, the most distressing symptoms supervene.

"The explanation seems to be this: As long as both auricles receive and discharge the same relative quantity of blood, no mixture to any extent can take place, even although the foramen ovale be very large; but, when the right becomes rather smaller, or of increased strength, or the left is seized with what is called passive aneurism, a portion of blood must go through to support the equilibrium. If the tricuspid valve, or the lungs themselves, become diseased, the same changes must happen.

"The above reasoning applies equally to cases where the septum of the ventricles is perforated. It seems probable, that, in our patient, the little variation that was found in the relative size of the heart was the cause of her surviving so long, and that death was produced from the adhesions of the lungs, and the increased strength of the right auricle: in the case also mentioned by Corvisart, the increased size of the right auricle, and other disease of the lesser circulation, were in all probability the causes of death."

This description, though apparently minute, is, we confess, not sufficiently so for us. This is not intended as a reproach to the author, because we are willing to admit that no one is sufficiently aware of inquiries which may be made by others on a subject familiar to himself. By the words "the lungs were adhering in most places," are we to understand that they merely adhered to the pleura, or that adhesion had taken place in the air-cells? If only the former, unless the adhesion were greater than the author's language would lead us to suppose, we cannot consider the cause equal to the effect; that is, we cannot consider the resistance the blood would meet with in its passage through the lungs as sufficient to cause any important change in the actions of the heart, nor even sufficient to produce the very great difficulty in breathing, or necessity of a constantly erect posture. We therefore suspect, that, besides the adhesions to the pleura, the lungs were consolidated by the adhesion of the air-cells, so that the blood could circulate with difficulty, and would be imperfectly oxygenated.

Let us now pursue our author's further arguments that "the foramen remaining open in advanced life, is admitted to be an unimportant matter by the ablest physiologists." This is one of the many subjects in which J. Hunter and Bichat have expressed themselves almost in the same words.

But Dr. Thomson's opinion is, that though, as long as the two auricles receive and discharge the same quantity of blood, this remaining aperture may be unimportant, yet the moment that equality ceases, the danger commences. We should rather say that the danger arises from this irregularity in the powers and capacities of the two auricles, or, perhaps, in the cause of that irregularity, than from the opening of the foramen. But a case of Dr. Marcet's,* to which our author refers, shows that this blue complexion may occur, not only without any disease in the heart, but without any remaining opening of the foramen. It is certainly highly probable, that, under certain circumstances of dyspnoea, the foramen, if slightly closed, may be opened afresh; but a cause sufficient to produce such an effect would, in our opinion, be sufficient to produce the blue complexion, with all the other fatal symptoms attending these unhappy cases.

Two instances of children follow, in one of which dissection proved that the foramen was open, and another which recovered. In the former the lungs were so much diseased as to account for the state of the heart on the principle we have advanced. In the other, the impediment to the circulation through the lungs being slighter, was probably overcome, and the foramen, being no longer necessary, soon closed. Some cases are quoted from other authors, in which the blueness was universal, yet the lungs only in fault; and the paper closes with some pathological conjectures, which it is not necessary for us to notice.

Art. III.—*Case of the Effects of Tobacco.* Communicated by MARSHALL HALL, M.D. &c.

In this city of London we are acquainted with so many old smokers who value themselves on never spitting whilst they smoke tobacco, that we cannot help doubting the inference drawn in the case before us. As the patient was relieved by bleeding and purging, we should impute the whole to apoplexy, a disease not at all uncommon among smokers and porter drinkers.

"Indeed the symptoms (says the author of the paper) of the case were sufficiently peculiar: Syncope, succeeded by nausea and vomiting, and subsequently by violent pain and affection of the head; coma, and stertorous breathing, without paralysis, and with little affection of the pulse; the tendency to dosing and to syncope on a change of posture, or on any exertion; the languor of the circulation observed in the extremities: all these circumstances, taken together, seem to characterize decidedly the effects of tobacco, and to distinguish them from any other affection. It may

* See our Journal, vol. xiv. p. 471.

be be useful, however, on similar occasions, to recall to mind, that apoplexy, an injury of the head, a fit of epilepsy, a state of deep intoxication, the effects of other narcotics, asphyxia, syncope, &c. are all affections which, with the one recorded in this case, require to be accurately distinguished from each other.

"The treatment employed by the surgeon in this case seems to have been very efficacious. Perhaps the opening of the jugular vein would have been equally so, as the lividity seemed to indicate a redundancy of venous blood. But in spasmodic affections, the temporal artery is more accessible, and more promptly and more conveniently opened. The particular treatment here mentioned was followed in the later periods of the complaint, by such remedies as the symptoms seemed to require, and especially by repeated doses of calomel."

The discrimination here recommended is important, and therefore we have taken the liberty of making the above remark.

Art. IV.—*Observations on the Plague at Malta.* By William Stafford, Surgeon, 1st, late the 3d, Garrison Battalion.

These observations are principally directed to the cure of that formidable disease. Mercury was the remedy chiefly relied on, with bark and wine in the more advanced stage. We respect the honesty and years of Mr. Stafford, and think it right, before we say more, to put in our claim to the same approach toward Nestorean prudence. But, as Sydenham lived to be older than either of us, we would remind our readers of his adage on the small-pox, namely, that there are cases which the physician cannot cure, and others which the nurses cannot kill.

A more important consideration is the means of prevention. On this Mr. S., with becoming modesty, gives great credit to the commanding officer.

"In detailing (says he) these cases, my intention was merely to relate facts, my mode of treatment, and the results; avoiding all theory or reasonings about it, knowing, that if you deem this paper of sufficient importance to merit a place in your valuable Journal, it will call the attention of men of the first abilities, who will do the subject more justice than I can. I am near sixty, and it is long since I was at school. This regiment was twice infected with plague; and, though it appeared in every company in it, you will be surprised to hear, that we lost on the whole only eleven men. It is not my intention to claim any merit on this account. That is entirely due to the commanding officer, Major C. Bayley, to whose almost sleepless zeal and attention for nine months it is owing that our loss was so small; to his wise regulations, and constant superintendance, the safety of a great part of the battalion must be owing."

Let

Let us now see what was the consequence where Major C. Bayley could not extend his cautions. In doing this, we shall return to that part of the history of each case which relates to such intercourse between the sick and the healthy as might lead to a communication of the disease.

"My battalion (says Mr. S.) was quartered at Floriana, a fortified outwork to the city of Valetta. The works are somewhat in the form of a half-moon, and are between two and three miles round. I imagine, at the commencement of the disease, it contained about 6000 souls, of which about 1600 died. Whole streets were depopulated, and very few houses escaped. *Under* my quarters, three separate families lived, and had no communication with each other, and some died out of each. Floriana is the only outlet from Valetta to the country. All the people that died in the city, all the sick sent into quarantine or observation, infected clothes, &c.—the dead carts, sometimes so full that the covers could not be shut down, and, from the pressure of the bodies on each other, the carts might be traced by the quantity of pest-matter running from them:—all the pest-hospitals for the Maltese, places of quarantine and observation, were in the ditches of Floriana, under the eyes of our sentries, who frequently, at day-break, saw heaps lying on each other dead; and, during the whole of the day, numbers under the severe and last agonies of the disease.

"I have been thus particular in describing the situation of Floriana, where ours was the only regiment to do every part of the military duty, by guarding all these extensive works. The officers and men had, of course, to traverse the town in every direction. I likewise wish to show how next to impossible it was that we could escape, as some severe observations have been made for our suffering it to appear among us. All the regiments quartered in Valetta were lodged in palaces. The door being locked, they might be supposed to be secure, yet some of them suffered, and one of them received the infection first."

We wish the reader who has not visited these kind of places to understand that by "families living *under* the author's quarters" is meant, that the basement story was occupied by families, whilst the rest of the palace formed the quarters for the military. Such was probably the case with the whole garrison of Valetta, who, we are informed, were all lodged in palaces.

The first case does not appear to have left the quarters; yet there is no suspicion annexed to the account that he spread the infection, though he continued ill from the 14th to the 18th, and was not discharged for duty till the 2d of the following month.

The second case is suspected to have happened "from some defect in the patient's shoe, in consequence of which some pest dust had got in." This is, however, only a conjecture.

The fourth case arose without any apparent cause, and though the subject continued with the regiment four days, it does not appear to have infected any person.

The fifth was of a man who slept with a woman during his complaint.

"This woman was sent into quarantine, but never betrayed the smallest symptom of the complaint.

"I was informed that a man-servant of Captain Chilcott, R. N., passed a night in a house of ill-fame. In a very short time after, a bubo appeared in the inferior gland of the groin, which, on inspection, was found to be plague. He was sent to the hospital, and died very soon after. I understand that this man did the usual duty of the house, and actually nursed one of the children a short time before he was sent to the hospital. The inspector of hospitals informed me, that a young lady in his neighbourhood married during the plague, and died soon after, and no other of the family. Sometime early in October, a serjeant of the 14th regiment, who acted as blacksmith to the officers of the garrison, had his forge in Floriana, and had one of our men to assist him. The serjeant being a very useful man, and excused all other duty, was allowed to live as he pleased, and to go when and where he pleased. He was very fond of liquor and women. It is known he frequently went into Valetta on pretences of business, and probably went into improper houses. He applied to a surgeon for relief for a tumour in the groin. It was examined, and supposed to be venereal, and intended to be treated as such, the man having no other apparent complaint at the time. On his being visited next morning, his case was found to be plague. He died the same evening. When inspected in the morning, the man belonging to our battalion was sleeping in the same bed with him. On examining the dead body, it was found to be a case of the most malignant kind, with large broad livid spots, and numberless petechiæ all over the trunk and thighs. As there was no doubt that our man had been in close contact with him, as they had constantly slept together, handled the same working tools, eating and drinking together, on placing him in observation, I was determined not to wait for the appearance of the symptoms, but try if I could prevent it. He was several times purged with calomel, and took small doses night and morning, until his mouth was sore, which happened soon. The same was continued. His mouth kept tender during the whole of his quarantine, 41 days. He had no complaint during the time, except once or twice a slight head-ach, which was always removed by a calomel purge. He had a generous diet all the time, and generally a pint of wine a-day."

We have been thus particular as to the progress of the supposed infection, and shall only say, that, if the disease was infectious, infectious, it was not in the same manner as the small-pox. There is no reason, to suppose that the preventive means adopted for the last subject were at all necessary or of use, if we may judge by the previous history in the same paragraph. In fine, we suspect that none but stragglers in this garrison were infected, and that, whatever intercourse took place, the disease was never communicated.

*(To be continued.)*

